# Lateral weight transfer deficits reveal balance vulnerability in early-stage Parkinson’s Disease during trip-perturbed walking

**DOI:** 10.21203/rs.3.rs-6298735/v1

**Published:** 2025-05-08

**Authors:** Anke Hua, Ruth Akinlosotu, Kelly Westlake

**Affiliations:** University of Maryland, Baltimore; University of Maryland, Baltimore; University of Maryland, Baltimore

**Keywords:** Parkinson’s Disease, Balance, Gait, Falls

## Abstract

**Background:**

Individuals with early-stage Parkinson’s disease (PD) typically exhibit normal balance during clinical assessments; but subtle impairments may exist during dynamic tasks.

**Objective:**

This study investigates reactive balance responses to trip perturbations during walking in early-stage PD compared to age-matched controls.

**Methods:**

Sixteen individuals with early-stage PD (Hoehn & Yahr 2–2.5) and sixteen age-matched controls walked on a treadmill, experiencing an unpredictable trip perturbation. Whole-body kinematics were analyzed to compute margin of stability (MoS) and lateral body center of mass (COM) displacement trajectories across four reactive steps. Statistical comparisons evaluated group differences in MoS and COM displacement, while correlation analyses assessed relationships between stability and lateral COM displacement.

**Results:**

By the third recovery step, controls had regained stability, while PD participants displayed significant variability. Half of the PD group exhibited negative MoS values, indicating instability, while the other half maintained stability comparable to controls. Moderate correlations between third-step MoS and lateral COM displacement (*r* > 0.56, *p* < 0.01) suggest impaired lateral weight transfer contributes to balance failure in PD.

**Conclusions:**

This study reveals variability in reactive balance capacity among early-stage PD participants, with nearly half showing subclinical deficits in lateral COM control. Trip-perturbed walking could serve as a promising biomarker for early balance impairments, potentially guiding proactive fall prevention strategies in PD management.

## 1. Introduction

Parkinson’s disease (PD) is a neurodegenerative disorder clinically characterized by a range of motor deficits that progressively worsen over time. The modified 7-point Hoehn and Yahr scale is widely used to classify the severity of motor symptoms in PD, with stages 1–2.5 represent the early stages of the disease. At these stages, balance is either clinically unaffected (stages 1–2) or shows mild impairment with recovery on the pull test (stage 2.5). However, emerging evidence suggests that subtle balance impairment may already be present in the early stages of PD,^1,2^ even when not detected through standard clinical assessments. This underscores the need to evaluate balance control during the early progression of PD.

Balance control is a multifaceted process, with reactive balance, the ability to recover stability following unexpected perturbations, being particularly critical for fall prevention ^3^. Individuals with early-stage PD have been shown to exhibit impaired reactive balance control from a static standing position, including reduced step length,^4^ increased center-of-mass (COM) acceleration, and prolonged recovery times after perturbations.^5^ Additionally, greater variability in balance responses has been observed in PD patients compared to age-matched controls across various perturbation types, such as external perturbations,^5^ visual disturbances,^6^ and self-initiated movements.^7^ This variability likely reflects distinct and inconsistent compensatory strategies among individuals with PD, pointing to underlying differences in balance control mechanisms.

While reactive balance impairments during standing have been well documented in early-stage PD, little is known about how these deficits manifest during dynamic activities like walking, an essential and higher risk task for falls. Walking requires continuous medio-lateral (ML) COM stability to maintain forward progression and execute fluent weight shifts between steps. Previous studies have shown that PD patients struggle to adequately shift body weight and control ML COM stability during tasks such as walking initiation,^8^ chair rising,^9^ turning,^10^ and repetitive stepping.^11^ Such difficulties are particularly pronounced in individuals experiencing freezing episodes and are often accompanied by reduced lateral COM displacement. These findings suggest that abnormalities in lateral shift of COM may play a pivotal role in reactive balance impairments in PD patients.

The present study aims to address this knowledge gap by investigating reactive balance control during trip-perturbed walking in individuals with early-stage PD compared to age-matched controls. Specifically, we hypothesized that: (1) participants with PD would exhibit reduced postural stability following a trip perturbation during walking compared to controls; (2) participants with PD would show greater variability in the number of steps needed to restore stability; and (3) postural stability after the perturbation will be positively associated with the efficiency of lateral COM displacement (i.e., amplitude and slope of peak-to-peak lateral COM displacement) during recovery steps. By examining these hypotheses, this study seeks to uncover key mechanisms underlying reactive balance impairments in early-stage PD and provide insights into potential biomarkers for early detection and intervention.

## 2. Methods

### Participants

This study included sixteen individuals diagnosed with Parkinson’s disease (PD) and sixteen age-matched neurotypical controls. Participant characteristics are detailed in Table 1.

Participants with PD were eligible if they met the following criteria: (1) Hoehn & Yahr stage 2–2.5, and (2) tested while “on” their regular dose of dopaminergic medication. Inclusion criteria for both groups required participants to be (1) 45 years of age or older, and (2) community ambulators capable of walking a 10-meter distance with or without a walking cane, corresponding to a score of 4 or greater on the Functional Ambulation Category scale. For the PD group, exclusion criteria included: (1) dyskinesias rated higher than grade 2 on the Unified Parkinson’s Disease Rating Scale, and (2) a history of brain surgery or deep brain stimulator placement. Exclusion criteria for both groups included: (1) cognitive impairment indicated by a score below 21/30 on the Montreal Cognitive Assessment (MoCA) or a Saint Louis University Mental Status (SLUMS) Exam score below 21 (or below 20 for those without a high school education), suggestive of dementia; (2) significant neurological, musculoskeletal, cardiovascular, or other impairments affecting activities of daily living (score less than 6/6 on the Katz Index); (3) clinically identified visual or hearing loss; (4) complaints of shortness of breath or uncontrolled pain exceeding 3/10 on a visual analog scale during rest or a five-minute walking bout; and (5) recent major surgery within six months or hospitalization within three months.

All participants provided written informed consent prior to participation. The study was conducted in a university laboratory setting, and all procedures were approved by the university’s institutional review board. Research adhered to the ethical principles outlined in the Declaration of Helsinki.

### Task and experimental set-up

The ActiveStep^®^ treadmill (ActiveStep, Simbex, Lebanon, NH, USA) was used to induce balance perturbations. Prior to the trial, participants walked for 80–120s on the treadmill to determine their self-selected walking speed. To minimize anticipation, the perturbation (a single trip) was delivered after two to three unperturbed walking trials. Participants were instructed to respond naturally if their balance was perturbed, then resume walking until the treadmill stopped.

The trip perturbation intensity was consistent with previous studies (1.5 m/s velocity, 0.15 cm displacement, 2.7m/s^2^ acceleration) ^12,13^ and was triggered during the single-limb support phase after 10 steps using the e-Trip function of the ActiveStep system. A safety harness was worn by all participants to prevent actual falls. A load cell integrated into the harness detected ‘in-task’ falls (≥ 30% body weight supported by the harness) and harness reliance (≥ 5%, but < 30% body weight supported), as defined in prior research.^14^

### Data collection and analysis

A whole-body plug-in gait model with 39 reflective markers was used to capture full-body kinematics, recorded by a 6-camera motion capture Vicon system (Vicon, Denver, CO) at a sampling frequency of 120 Hz. Markers were placed on bilateral upper and lower extremities, trunk, and head to compute joint centers and center of mass (COM).^15^ Kinematic variables were processed using a custom-written MATLAB algorithm (R2021a, MathWorks, USA). The raw marker data were low-pass filtered using a second order Butterworth filter with a 10 Hz cutoff.^16^

Since the trip perturbation induced postural instability primarily in the anterior-posterior (AP) direction, the AP margin of stability (MoS) was selected as the primary outcome measure for evaluating reactive balance responses during walking. AP MoS was calculated at the instant of protective step touchdown (defined as the lowest position of the heel marker) for each of four consecutive steps following the trip perturbation. Additionally, step response time (defined as the duration between trip perturbation onset and step touch-down) was calculated for each of these four steps.

1 $$\mathrm{Margin}\;\mathrm{of}\;\mathrm{Stability}\;=\;\mathrm{Margin}\;-\left(COM_{\mathrm{pos}}+\frac{COM_{\mathrm{vel}}}{\sqrt{\frac{g}{l}}}\right).$$

where g= gravity (9.8 m/s^2^); I= length between COM and foot;^17^ COMpos = vertical projection of COM on the ground; COMvel= instantaneous COM velocity in AP direction; Margin= distance between instantaneous COM position in AP direction and the anterior boundary of base of support (BoS) (i.e., feet’s 2nd metatarsal head).^18^

Secondary outcomes included the amplitude and slope of peak-to-peak lateral COM displacement following the trip perturbation to quantify the efficiency of lateral COM shifts during balance recovery after a trip.

### Statistical analysis

A two-way mixed ANOVA was conducted to assess the effects of group (PD vs. control) and step (1st, 2nd, 3rd, and 4th steps) on both the MoS and the step response following the trip perturbation. Post-hoc comparisons were performed with Bonferroni correction. A one-way ANOVA was used to evaluate group effects on the amplitude and slope of the peak-to-peak lateral COM displacement, with Bonferroni-adjusted post-hoc tests. Finally, two-tailed Pearson’s correlation analyses were conducted to examine the relationship between MoS and lateral COM shift efficiency (as measured by amplitude and slope), with Bonferroni correction applied for multiple comparisons.

## 3. Results

### Group Difference in Margin of Stability after Trip Perturbation

**Figure 1:**
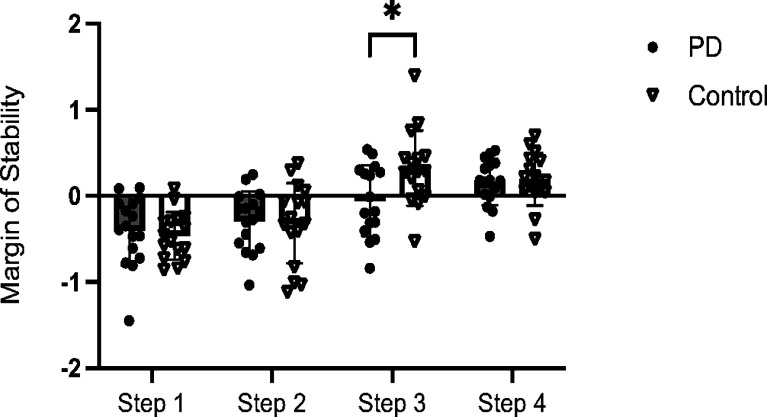
Margin of Stability Across Steps Following a Walking Trip Perturbation. Margin of stability (MoS) across four recovery steps after the trip perturbation for participants with Parkinson’s Disease (PD) and age-matched control group (Control). Mean values and standard deviations (SD) are displayed, along with individual data points for each participant. The asterisk (*) denotes a significant difference (*p* < 0.05) between the PD and Control groups.

During the first step following the trip perturbation, both the PD and control groups exhibited a negative MoS, suggesting postural instability in the AP direction. A two-way mixed ANOVA (group× step) revealed that MoS progressively increased across steps (step effect: *F*_*(3,90)*_ = 37.18, *p* < 0.001, *η^2^*=0.55) in both groups, with a more pronounced improvement observed in the control group (step × group interaction: *F*_*(3,90)*_ = 3.92, *p* = 0.01, *η^2^*=0.12). Post-hoc analysis with Bonferroni correction revealed that the MoS during the third step was significantly smaller in the PD group compared to the control group (*p* = 0.02) (Fig. 1). In contrast, a two-way mixed ANOVA did not reveal a significant group effect on step response time after trip perturbation (*F*_*(3,90)*_ = 0.61, *p* = 0.44).

Given the observed group difference in third step MoS and high variability within the PD group, participants were further categorized into three subgroups for subsequent analysis: the control group, PD participants with a positive third-step MoS (PD( + )) and PD participants with negative third-step MoS (PD ( − )).

### Group Differences in Lateral COM Transfer Efficiency after Trip Perturbation

**Figure 2:**
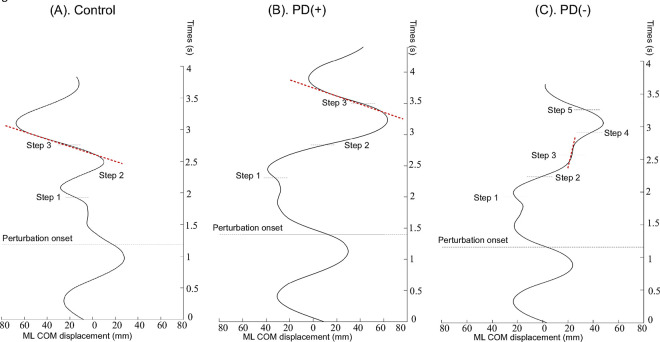
Characteristics of Center of Mass (COM) displacements in the Mediolateral (ML) direction Following a Walking Trip-Perturbation (A). ML COM displacement in a representative age-matched control participant with positive margin of stability (MoS) at the third step touchdown after the trip perturbation. (B). ML COM displacement in a representative individual with Parkinson disease (PD) who also demonstrated a positive MoS at the third step touchdown after the perturbation. (C). ML COM displacement in a representative individual with PD who exhibited negative MoS at the third step touchdown after the perturbation. The red dotted lines indicate the slope, representing the rate of change in peak-to-peak ML COM displacement during the third reactive step.

To quantify the efficiency of lateral COM shifts during recovery, the amplitude and slope of peak-to-peak lateral COM displacement were calculated for the third recovery step following the trip perturbation. Figure 2 illustrates the characteristics of COM displacements during third step in the mediolateral (ML) direction during trip-perturbed walking in a representative participant from the control, PD(+), and PD(−) groups.

**Figure 3:**
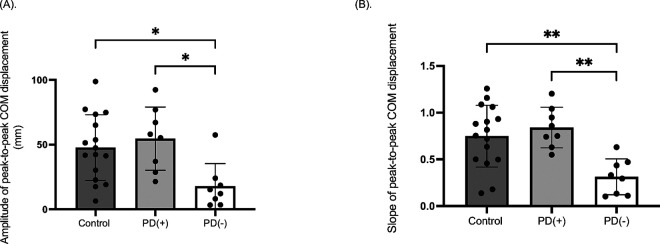
Group Differences in Lateral COM displacement Following a Walking Trip Perturbation. (A) Amplitude of peak-to-peak lateral center of mass (COM) displacement. (B) Slope of peak-to-peak lateral COM displacement. Mean values and standard deviations (SD) are presented, along with individual data points for each participant. Asterisks (*) indicate statistically significant difference (*p<0.05, **p<0.01). Groups include the age-matched control group (Control); participants with Parkinson disease (PD) who demonstrated a positive margin of stability at the third recovery step (PD(+)), and participants with PD who demonstrated a negative margin of stability at the third step (PD(−)).

A one-way ANOVA revealed significant group effects for both the amplitude (*F*_*(2,29)*_ = 6.90, *p* = 0.003, *η^2^*=0.33) and the slope (*F*_*(2,29)*_ = 10.78, *p* < 0.001, *η^2^*=0.43) of peak-to-peak COM displacement. Post-hoc analysis with Bonferroni correction showed that both the amplitude and slope of lateral COM displacement were significantly smaller in the PD( − ) group compared to both the PD( + ) and control group (*p* < 0.05) (Fig. 3).

### Correlations Between Margin of Stability and Lateral COM Transfer Efficiency

**Figure 4:**
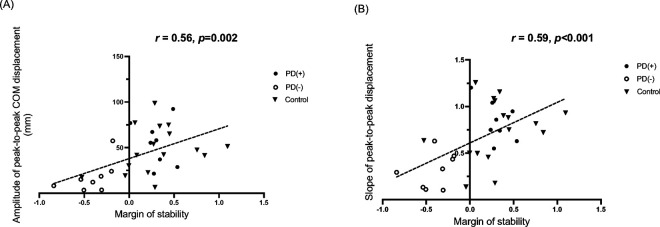
Correlations between Margin of Stability and Lateral COM Displacement Efficiency Following a Walking Trip Perturbation (A) Relationship between third recovery step margin of stability (MoS) (x-axis) and the amplitude of peak-to-peak lateral COM displacement (y-axis) during the third recovery step following the perturbation. (B) Relationship between the third recovery step MoS (x-axis) and the slope of peak-to-peak lateral COM displacement (y-axis) during the third recovery step following the perturbation. Black triangles represent participants in the age-matched control group (Control); black circles represent participants with Parkinson disease (PD) who had a positive MoS at the third step (PD(+)); and white circles represent participants with PD who had a negative MoS at the third step (PD(−)).

Significant positive correlations were observed between the third recovery step MoS and two measures of lateral COM transfer efficiency. Specifically, the amplitude of peak-to-peak lateral COM displacement was positively correlated with the third-step MoS (*r* = 0.56, *adjusted p* = 0.002) (Fig. 4A). Similarly, the slope of peak-to-peak lateral COM displacement during the third step also showed a significant positive correlation with the third step MoS (*r* = 0.59, *adjusted p* < 0.001) (Fig. 4B).

## 4. Discussion

The present study provides novel insights into the biomechanical mechanisms underlying reactive balance impairments during perturbed walking in early-stage PD (Hoehn & Yahr 2–2.5). These findings extend prior work on reactive responses from static standing into the dynamic realm of gait, demonstrating that 50% of the PD participants exhibited instability beyond the third recovery step following a trip perturbation, which was linked to diminished amplitude (65% reduction) and rate of change (60% reduction) of lateral center of mass (COM) displacement. The correlation between lateral COM shift efficiency and margin of stability (MoS) (*r* = 0.56–0.59) underscores the critical role of medio-lateral weight transfer in balance recovery, a mechanism previously implicated in uninterrupted turning^10^ and repetitive stepping in place,^11^ but not systematically examined during perturbed walking in individuals with PD.

### Impaired Lateral COM Control and Instability

The observed deficits in lateral COM control (Fig. 3) align with broader evidence of axial instability in PD.^4,5,19,20^ During walking, individuals must continuously adjust their COM trajectory to maintain stability, a task requiring coordinated trunk rotation and limb placement. Our finding that inefficient lateral shifts is related to instability (Fig. 4) mirrors patterns seen during freezing episodes, where restricted trunk motion exacerbates balance loss.^21,22^ This mechanistic overlap suggests that reactive balance impairments during perturbations (Fig. 1) and spontaneous gait freezing may share common substrates, potentially rooted in basal ganglia-mediated disruptions to intersegmental coordination.^24^

Notably, the high interindividual variability in PD responses (Fig. 2) parallels findings from gait adaptability studies, where executive dysfunction and slowed reaction times disproportionately affect stepping accuracy in PD.^25,26^ While our protocol did not directly assess cognitive contributions, the stratification of PD participants into “stable” and “unstable” subgroups raises the possibility that compensatory cognitive strategies, such as attentional focus on lateral weight shifts, may have enabled some individuals to maintain stability despite disease-related motor deficits. This hypothesis is supported by dual-task studies demonstrating that conscious control strategies can transiently ameliorate gait impairments in early PD.^27^

### Mechanisms of Impaired Lateral COM Control

The attenuated lateral COM displacement in the PD(−) subgroup likely reflects dopaminergic dysfunction in circuits coordinating anticipatory postural adjustments.^28^ The basal ganglia’s role in scaling movement amplitude may be particularly critical for generating sufficient lateral momentum during compensatory steps. Animal models of PD demonstrate impaired subthalamic nucleus modulation during locomotion, a deficit reversible with dopaminergic therapy.^29^ However, the persistence of lateral COM impairments in our medicated cohort suggests that non-dopaminergic pathways, possibly involving cholinergic system degeneration, contribute to these abnormalities.

The correlation between lateral COM metrics and MoS (Fig. 4) implies that effective weight transfer facilitates forward progression arrest by optimizing the base of support. This complements computational models^30^ showing that lateral stability margins dictate the success of anterior-posterior recovery strategies during trips. In PD, rigidity and bradykinesia may limit the rapid hip abductor activation needed for adequate COM displacement, increasing reliance on ineffective multistep recovery patterns.

### Clinical Implications for Fall Risk Assessment

Our results advocate for incorporating dynamic perturbation-based assessments into early PD evaluations. Traditional clinical tests like the pull test fail to capture the 50% failure rate in trip recovery observed here, supporting the use of instrumented gait analysis to detect subclinical deficits. The stratification of PD participants based on third-step MoS provides a potential biomarker for fall risk stratification, with the PD(−) subgroup likely representing a high-risk phenotype warranting targeted intervention.

Rehabilitation strategies should emphasize lateral weight-shifting capacity,^31^ as suggested by the strong association between COM displacement metrics and stability following a walking perturbation. Perturbation-based treadmill training, shown to improve reactive stepping in PD,^32^ could be adapted to specifically challenge lateral stability through medio-lateral platform translations. Combining this with cognitive-motor training targeting executive function may address the interplay between motor and cognitive deficits contributing to balance failures.

### Methodological Considerations and Future Directions

While this study advances understanding of reactive balance in PD, several limitations merit attention. The exclusion of PD participants with freezing of gait (FOG) precludes generalization to this high-risk subgroup. Given that FOG is associated with exaggerated lateral COM oscillations during repetitive stepping in place^11^ and turning,^10^ future work should examine how perturbation responses differ across PD motor subtypes. Additionally, the single-assessment design cannot determine whether lateral COM impairments represent compensatory strategies or primary deficits. Longitudinal tracking could clarify their prognostic value.

The lack of medication-OFF assessments leaves open questions about dopaminergic responsiveness of lateral COM control. Prior work shows levodopa medication did not significantly improve reactive stepping responses to multidirectional perturbations,^33,34^ suggesting our findings may persist across medication states. However, cholinergic interventions enhancing attention and sensory integration could differentially benefit subgroups, an avenue requiring exploration.

## Conclusion

By elucidating the critical role of lateral COM control in walking trip recovery, this study redefines early PD balance impairment as a dynamic, task-specific deficit rather than a global postural instability. The bifurcation of PD participants into stable and unstable subgroups underscores the heterogeneity of balance compensation strategies, necessitating personalized rehabilitation approaches. Integrating perturbation-based assessments into clinical practice could enable earlier identification of fall-prone individuals, while targeted training of lateral weight shifts may mitigate fall risk, a hypothesis awaiting validation in randomized trials. These findings position lateral COM regulation as a central therapeutic target in PD balance rehabilitation, bridging the gap between laboratory-based biomechanics and real-world fall prevention.

## Figures and Tables

**Table 1:** Participant characteristics

Characteristic	PD group (n = 16)	Control group (n = 16)	*p-value*

Sex (female:male)	2:14	8:8	0.64
Age (years)	70.12 ± 6.68	69.12 ± 5.03	0.29
BMI	25.84 ± 4.74	27.69 ± 4.50	

ABC score (%)	87.09 ± 5.97	91.55 ± 9.48	0.13
TUG (s)	8.66 ± 1.92	7.93 ± 1.71	0.28

PD, Parkinson disease; ABC, Activities-specific Balance Confidence scale; TUG, Timed Up and Go test; H & Y stage, Hoehn and Yahr stage. Values are reported in mean ± standard deviation.

## Data Availability

The data supporting the findings of this study are available on request from the corresponding author. The data are not publicly available due to privacy or ethical restrictions.
